# Infective endocarditis in a Finnish tertiary care hospital: from etiology to embolic events

**DOI:** 10.1080/07853890.2024.2415405

**Published:** 2024-11-09

**Authors:** Elina Ahtela, Ville Kytö, Tero Vahlberg, Ulla Hohenthal, Tommi Ekström, Pekka Porela, Jarmo Oksi

**Affiliations:** a Heart Center, Turku University Hospital and University of Turku, Turku, Finland; b Department of Infectious Diseases, Turku University Hospital and University of Turku, Turku, Finland; c Turku Clinical Research Center, University of Turku, Turku, Finland; d Department of Clinical Medicine, Biostatistics, University of Turku and Turku University Hospital, Turku, Finland

**Keywords:** Infective endocarditis, etiology, embolic event, cerebral embolism

## Abstract

**Background:**

In this study in a tertiary care hospital, we examined the characteristics of the different microbial etiologies of infective endocarditis (IE) and the factors associated with embolic events.

**Materials and methods:**

We included patients (aged ≥18 years) hospitalized for IE in Turku University Hospital in Finland between 2004-2017. Patient data were derived retrospectively from the mandatory database and patient record system.

**Results:**

Among 342 IE cases in 321 patients, *Staphylococcus aureus* was isolated in 33.9%, viridans group streptococci in 18.3% and enterococci in 8.8% of the cases. Patients with enterococcal IE had more often a prosthetic valve (*p* < 0.001), recent major healthcare procedure or hospital admission (*p* < 0.001) and heart failure during admission (*p* = 0.006) than the patients with other etiologies. Viridans group streptococci and enterococci vs. *S. aureus* were associated with a lower rate (OR 0.34, *p* = 0.007 and OR 0.20, *p* = 0.006, respectively) and IE of the multiple valves vs. aortic valve with a higher rate (OR 2.30, *p* = 0.043) of all embolic events but not cerebral embolisms when analyzed separately. Both all embolic events and cerebral embolisms were strongly associated with the occurrence of an echocardiography-disclosed vegetation (OR 3.31, *p* = 0.004 and OR 2.73, *p* = 0.019, respectively).

**Conclusions:**

Our study suggests that enterococcal IE is often associated with a previous healthcare procedure or hospital admission and heart failure. *Staphylococcus aureus* etiology and IE of the multiple valves are associated with a higher rate of all embolic events but not cerebral embolisms. Echocardiography-disclosed vegetation is associated with a higher occurrence of embolisms.

## Introduction

Infective endocarditis (IE) is a severe disease and is associated with serious complications. The profile of IE has changed remarkably over the recent years due to advanced diagnostic and therapeutic methods, aging of the population and changed risk factors for IE [1]. The proportion of healthcare-associated IE is increasing and is nowadays around 30% of all IE cases [2–8]. The most common causative agent nowadays is *Staphylococcus aureus* accounting for up to 30% of IE cases [6,9–12]. *Staphylococcus aureus* has been found to be the major causative microbe in healthcare-related IE [3,4,10] and furthermore in intravenous drug users’ (IVDUs’) IE [8,13–16]. However, especially in healthcare-related IE, enterococci have emerged as a noteworthy etiology [3,5,8,17–19]. Nevertheless, up-to-date data comparing the characteristics of the different microbiological etiologies of IE are needed.

Embolic events are common complications of IE occurring in 34-55% of IE cases [7,10,11,20–22]. Cerebral embolisms are usually seen in left-sided IE, whereas pulmonary embolisms occur in right-sided IE [1]. *Staphylococcus aureus* etiology has been reported to be associated with an increased risk of embolic events in IE [6,21–24]. However, contradictory findings have been made about the association of the affected heart valve with the occurrence of the embolic events. Several studies did not find any association of the valve localization with embolic events [20,22,25–30]. Some studies found mitral valve IE to be associated with a higher rate of embolic events [31,32], while right-sided [6,21] and aortic valve IE [33] have also been reported to be predictors of embolic events. Furthermore, an infected mitral valve has been found to be associated with cerebral complications of IE [34–39]. Nevertheless, some studies reported no difference in the valve involvement of IE in the occurrence of cerebral complications [40,41].

We studied the characteristics of the different microbiological etiologies causing IE and the factors, especially the microbial etiology and valve involvement, associated with all embolic events and specifically cerebral embolisms.

## Materials and methods

### Study design and data collection

We included patients (≥18 years of age) hospitalized for IE in Turku University Hospital in Finland, Hospital District of Southwest Finland, between January 1, 2004, and June 31, 2017. The hospital has a cardiothoracic surgical unit and serves as a tertiary referral center in southwestern part of Finland, covering a population of approximately 870 000 individuals. Patients were recognized retrospectively according to the discharge diagnosis of IE (International Classification of Diseases, Tenth revision (ICD-10) codes I33, I38 and I39) from the hospital’s patient record archive. The study was approved by the Hospital District of Southwest Finland (permission no. TO2/015/17). The legal basis for processing of personal data is public interest and scientific research (EU General Data Protection Regulation 2016/679 (GDPR), Article 6(1)(e) and Article 9(2)(j); Data Protection Act, Sections 4 and 6). Patient consent was waived by the applicable Finnish law due to the retrospective study design. Results from this paper have been presented to the University of Turku as a thesis with interim findings [42]. The thesis is available on the institution’s repository https://urn.fi/URN:ISBN:978-951-29-9102-0.

### Definitions

Patients with definite or possible diagnosis of IE according to the modified Duke criteria and the 2015 European Society of Cardiology (ESC) guidelines for the management of IE [1,43] were included in the study. We investigated 342 IE cases in 321 patients. In three IE cases, IE could not be specified to any heart valve (two with a non-specific mass inside the atrium of the heart and one with a ventricular septal defect), and they were excluded. Microbial etiology of IE was determined from the blood cultures and bacterial culture and polymerase chain reaction (PCR) from the resected heart valve. Bactec blood culture systems (BD, Franklin Lakes, NJ, US) were used throughout the study period: Bactec 9240 and 9050 automates during 2004-2012 and Bactec Fx from 2013 onwards. Each blood culture set contained both an aerobic and an anaerobic flask validated at the time. The bacterial growth in positive flasks was identified by a standard gram stain, followed by species identification with use of VITEK2 system (bioMerieux, Marcy l’Etoile, France) until 2011 and by Maldi-TOF MS (matrix-assisted laser-desorption ionization time-of-flight mass spectrometry) instruments (Bruker, Bremen, Germany) since 2011. Biopsies of resected heart valves were homogenized and inoculated on standard blood, chocolate and FAA (fastidious anaerobe) agars, as well as liquid medium (fastidious anaerobe broth) and incubated for 14 days. The growth was identified using standard methods described above, and 16S ribosomal ribonucleic acid (rRNA) PCR on valve biopsies was performed as described previously [44].

Embolic events were defined as all embolic events, cerebral embolisms and pulmonary embolisms. All embolic events included both cerebral and pulmonary embolisms. Embolic events were diagnosed by computed tomography (CT), magnetic resonance imaging (MRI), positron emission tomography integrated with computed tomography (PET/CT) or clinically. Patient was defined to have an embolic event if the diagnosis (cerebral embolism, embolism of a specific organ, e.g. spleen, kidney, liver or lungs) was derived from the imaging or from a clinical examination of the clinician (signs of an embolism in the skin or cerebral embolism in the case when imaging was not performed, e.g. due to the patient′s condition). For some of the IE cases, certain collected information was missing in the patient record, for example as a consequence of a transfer from another hospital. These cases were excluded from the analyses requiring the missing information. The number of the cases with missing information are mentioned in the tables alongside the particular parameter or in the text.

### Statistics

Patient characteristics and the factors associated with the different etiologies and embolic events were analyzed with the Chi-squared test, independent samples t-test and Kruskal-Wallis one-way analysis of variance. Normality was tested visually using histograms. For the normally distributed factors mean and standard deviation (SD) were presented and for the non-normally distributed median and interquartile range (IQR). Logistic regression using generalized estimating equations was used to analyze the factors associated with embolic events to account for the possible correlations between the values of the different admissions of the same patients. In addition to the age group and sex, the variables with p-value <0.1 in the univariable analysis were included in the multivariable models. The correlations between the clinical factors in the multivariable analysis were studied with Phi coefficient in crosstabulation to avoid multicollinearity problems, and values lower than 0.7 were included. Results are represented as odds ratio (OR) with 95% confidence interval (CI). P-values less than 0.05 were considered statistically significant. Two-sided p-values were used. Statistical analyses were performed with the SPSS version 28 (Armonk, NY: IBM Corp.).

## Results

### Patient characteristics

There were 342 IE cases in 321 patients during the study period. The characteristics of the cases are presented in Table 1. Majority of the cases were in men (75.1%). The mean age of the patients was 59.0 years (SD 19.1, range 18-97). There was no difference in the mean age between the sexes (women 60.7 years, SD 22.4 vs. men 58.4 years, SD 17.9; *p* = 0.379). Intravenous drug users consisted of 18.5% of the patients. The mean age of the IVDUs was 30.4 years (SD 6.4, range 18-46) and 69.8% were men. Of the patients, 18.1% had a prosthetic valve. The number of patients transferred to Turku University Hospital from outside of the Hospital District of Southwest Finland due to the severity of IE was 35.

**Table 1.** Characteristics of the cases of infective endocarditis (*n* = 342) in Turku University Hospital in 2004-2017.

	No of cases (%)
Sex	
Female	85 (24.9)
Male	257 (75.1)
Age group	
18-39 years	78 (22.8)
40-59 years	77 (22.5)
60-79 years	141 (41.2)
≥80 years	46 (13.5)
IVDU	
Yes	63 (18.5)
No	278 (81.5)
Previous anticoagulation therapy	
Yes	85 (24.9)
No	256 (75.1)
Coronary artery disease	
Yes	57 (16.7)
No	285 (83.3)
Diabetes	
Yes	68 (19.9)
No	274 (80.1)
Chronic dialysis	
Yes	17 (5.0)
No	324 (95.0)
Prosthetic valve	
Yes	62 (18.1)
No	280 (81.9)
CIED	
Yes	21 (6.1)
No	321 (93.9)
Previous IE	
Yes	23 (6.7)
No	319 (93.3)
Major healthcare procedure or hospital admission during 6 months before admission	
Yes	128 (41.3)
No	182 (58.7)
Diagnosis according to modified Duke criteria and ESC guidelines 2015	
Definite	231 (67.5)
Possible	111 (32.5)
Etiology	
*Staphylococcus aureus*	115 (33.9)
Viridans group streptococci	62 (18.3)
Enterococci	30 (8.8)
Coagulase-negative staphylococci	24 (7.1)
Other^a^	67 (19.8)
Unknown	41 (12.1)
Echocardiography	
Transthoracic	340 (99.4)
Transesophageal	246 (73.4)
CT or MRI of the brain performed	
Yes	128 (40.0)
No	192 (60.0)
Other CT or MRI performed	
Yes	174 (54.5)
No	145 (45.5)
PET/CT performed	
Yes	45 (13.2)
No	296 (86.8)
Affected valve	
Aortic only	156 (45.6)
Mitral only	95 (27.8)
Tricuspid only	46 (13.5)
Multiple valves	45 (13.2)
Vegetation on echocardiography	
Yes	257 (75.8)
No	82 (24.2)
Heart failure during admission	
Yes	177 (53.8)
No	152 (46.2)
Valve surgery performed	
Yes	90 (26.5)
No	250 (73.5)

CIED: Cardiac implantable electronic device; CT: Computed tomography; ESC: European Society of Cardiology; IE: Infective endocarditis; IVDU: Intravenous drug use; MRI: Magnetic resonance imaging; PET/CT: Positron emission tomography integrated with computed tomography.

^a^Other microbe than *Staphylococcus aureus*, viridans group streptococcus, enterococcus, coagulase-negative staphylococcus; or two or more microbes from the different groups mentioned.

Of the IE cases, a significant proportion (41.3%) had a history of a major healthcare procedure (cardiovascular, gastrointestinal, urological or other invasive procedure) or hospital admission during 6 months or a central venous catheter (not dialysis-associated) during one month before IE admission.

Transthoracic echocardiography (TTE) had been performed in virtually all patients (99.4%) and transesophageal echocardiography (TEE) in 73.4%. Infective endocarditis of the aortic valve was the most common accounting for 45.6% of the cases, whereas mitral valve IE consisted of 27.8% of the cases. Tricuspid valve was infected in 13.5% of all IE cases and in 57.1% of the IVDUs’ IEs. A vegetation was detected on echocardiography in 75.8% of the cases. Of the cases, 67.5% had definite diagnosis of IE according to the modified Duke criteria and the 2015 ESC guidelines.

### Etiology

Etiology was determined in 87.9% of the IE cases. *Staphylococcus aureus* was the most common etiology of IE and accounted for 33.9% (*n* = 115) of IE cases. Viridans group streptococci were the second most common etiology and consisted of 18.3% (*n* = 62) of cases, whereas enterococci (8.8%, *n* = 30) were third. Blood cultures were negative in 13.9% (*n* = 47) of the cases. Of the blood culture-negative cases, etiology was determined in 6 cases: in 5 cases with bacterial culture from the resected valve and in 1 case with PCR from the resected valve. All microbes detected are listed in Table 2.

**Table 2.** Microbial etiology in 339 cases of infective endocarditis in Turku University Hospital in 2004-2017. Percentages counted of the total number of microbes found (*n* = 339).

Microbe		N (%)
*Staphylococcus aureus*		124 (36.6)
Methicillin-susceptible		123
Methicillin-resistant		1
Viridans group streptococci^a^		78 (23.0)
Enterococci		33 (9.7)
*Enterococcus faecalis*		30
*Enterococcus faecium*		3
Coagulase-negative staphylococci^b^		35 (10.3)
Beta-hemolytic streptococci		19 (5.6)
*Streptococcus agalactiae*		6
*Streptococcus dysgalactiae*		5
*Streptococcus dysgalactiae subspecies equisimilis*		1
*Streptococcus pyogenes*		2
Not specified		5
*Streptococcus pneumoniae*		7 (2.1)
*Escherichia coli*		6 (1.8)
*Bacillus cereus*		4 (1.2)
*Candida albicans*		4 (1.2)
Other^c^		29 (8.6)
Polymicrobial		32
Unknown etiology		41

^a^*S. mitis 23, S. sanguinis 11, S. gordonii 9, S. oralis 8, S. mutans 6, S. anginosus 3, S. gallolyticus 3, S. constellatus 2, S. equinus 2, S. salivarius 3, S. bovis 1, S. parasanguinis 1, S. gallinaceus 1, not specified 5*.

^b^*S. epidermidis 15, S. hominis 6, S. lugdunensis 5, S. warneri 5, S. capitis 1, S. haemolyticus 1, not specified 2*.

^c^*Aerococcus urinae 3, Bartonella quintana 1, Corynebacterium jeikeium 1, Granulicatella adiacens 2, Enterobacter cloacae 2, Serratia marcescens 2, Abiotrophia defectiva 1, Acinetobacter pittii 1, Capnocytophaga canimorsus 1, Eggerthella lenta 1, Gemella morbillorum 1, Klebsiella pneumoniae 1, Lactobacillus rhamnosus 1, Leclercia adecarboxylata 1, Propionibacterium/Cutibacterium acnes 4, Propionibacterium species 1, Pseudomonas fulva 1, Salmonella muenchen 1, Serratia ficaria 1, Sphingomonas paucimobilis 1, Stenotrophomonas maltophilia 1*.

Factors associated with the different etiologies are presented in Table 3. Patients with *S. aureus* IE were the youngest (median age 53.0 years), whereas the oldest were the patients with enterococcal IE (median age 74.5) (*p* < 0.001 for overall comparison). In *S. aureus* IE and IE caused by the etiological group Other (other microbe than *S. aureus*, viridans group streptococcus, enterococcus, coagulase-negative staphylococcus; or two or more microbes from the different groups mentioned), patients were more often IVDUs (35.7% and 19.4%) than the patients with other etiologies (*p* < 0.001 for overall comparison). Of IVDUs, 66.1% had *S. aureus* IE.

**Table 3.** Factors associated with the different etiologies^a^ in cases of infective endocarditis (*n* = 339) in Turku University Hospital in 2004-2017.

		*Staphylococcus aureus* (*n* = 115) (%)	Viridans group streptococci (*n* = 62) (%)	Enterococci (*n* = 30) (%)	Coagulase-negative staphylococci (*n* = 24) (%)	Other^b^ (*n* = 67) (%)	Unknown (*n* = 41) (%)	P
Sex								
Female (*n* = 84)		32 (27.8)	12 (19.4)	6 (20.0)	7 (29.2)	14 (20.9)	13 (31.7)	0.584
Male (*n* = 255)		83 (72.2)	50 (80.6)	24 (80.0)	17 (70.8)	53 (79.1)	28 (68.3)	
Median age (IQR)		53.0 (37)	62.0 (26)	74.5 (13)	70.0 (18)	60.0 (35)	67.0 (17)	<0.001
IVDU^c^								
Yes (*n* = 62)		41 (35.7)	0 (0.0)	4 (13.3)	1 (4.2)	13 (19.4)	3 (7.5)	<0.001
No (*n* = 276)		74 (64.3)	62 (100)	26 (86.7)	23 (95.8)	54 (80.6)	37 (92.5)	
Coronary artery disease								
Yes (*n* = 57)		17 (14.8)	8 (12.9)	5 (16.7)	8 (33.3)	10 (14.9)	9 (22.0)	0.248
No (*n* = 282)		98 (85.2)	54 (87.1)	25 (83.3)	16 (66.7)	57 (85.1)	32 (78.0)	
Diabetes								
Yes (*n* = 68)		24 (20.9)	10 (16.1)	7 (23.3)	7 (29.2)	15 (22.4)	5 (12.2)	0.565
No (*n* = 271)		91 (79.1)	52 (83.9)	23 (76.7)	17 (70.8)	52 (77.6)	36 (87.8)	
Prosthetic valve								
Yes (*n* = 60)		10 (8.7)	5 (8.1)	13 (43.3)	9 (37.5)	16 (23.9)	7 (17.1)	<0.001
No (*n* = 279)		105 (91.3)	57 (91.9)	17 (56.7)	15 (62.5)	51 (76.1)	34 (82.9)	
Major healthcare procedure or hospital admission during 6 months before admission^d^								
Yes (*n* = 126)		36 (35.6)	13 (23.2)	19 (67.9)	15 (62.5)	20 (32.8)	23 (62.2)	<0.001
No (*n* = 181)		65 (64.4)	43 (76.8)	9 (32.1)	9 (37.5)	41 (67.2)	14 (37.8)	
Affected valve								<0.001
Aortic only (*n* = 156)		35 (30.4)	32 (51.6)	16 (53.3)	18 (75.0)	33 (49.3)	22 (53.7)	
Mitral only (*n* = 94)		30 (26.1)	23 (37.1)	10 (33.3)	3 (12.5)	14 (20.9)	14 (34.1)	
Tricuspid only (*n* = 46)		32 (27.8)	0 (0.0)	1 (3.3)	1 (4.2)	9 (13.4)	3 (7.3)	
Multiple valves (*n* = 43)		18 (15.7)	7 (11.3)	3 (10.0)	2 (8.3)	11 (16.4)	2 (4.9)	
Heart failure during admission^e^								
Yes (*n* = 176)		54 (49.1)	22 (36.7)	20 (74.1)	16 (66.7)	39 (60.0)	25 (62.5)	0.006
No (*n* = 150)		56 (50.9)	38 (63.3)	7 (25.9)	8 (33.3)	26 (40.0)	15 (37.5)	

IQR: Interquartile range; IVDU: Intravenous drug use.

^a^Cases with missing information: 3.

^b^Other microbe than *Staphylococcus aureus*, viridans group streptococcus, enterococcus, coagulase-negative staphylococcus; or two or more microbes from the different groups mentioned.

^c^Cases with missing information: 1.

^d^Cases with missing information: 32.

^e^Cases with missing information: 13.

Patients with enterococcal IE more often had a prosthetic valve (43.3%) than the patients with other etiologies (*p* < 0.001 for overall comparison). Only 8.7% of the patients with *S. aureus* IE had a prosthetic valve. In the overall comparison, there was no difference in the prevalence of coronary artery disease or diabetes between the different etiologies. Enterococcal IE patients had more frequently had a previous healthcare procedure or hospital admission (67.9%) than the patients with other etiologies (*p* < 0.001 for overall comparison). Of the patients with *S. aureus* IE, 35.6% had a history of a previous healthcare procedure or hospital admission. Of all patients with a previous healthcare procedure or hospital admission, 28.6% had *S. aureus* IE and 15.1% enterococcal IE.

Tricuspid valve was more commonly affected (27.8%) in IE caused by *S. aureus* than in IE caused by other etiologies (*p* < 0.001 for overall comparison). Of the different etiologies, heart failure during IE admission was the most frequent in enterococcal IE (74.1%) and the least common in IE caused by viridans group streptococci (36.7%) (*p* = 0.006 for overall comparison).

### All embolic events

During the IE admission, 125 (39.1%) patients were diagnosed with an embolic event (Table 4). In the univariable analysis, the factors associated with all embolic events were the youngest age group vs. other age groups, intravenous drug use (IVDU), not having previous anticoagulation therapy, not having coronary artery disease or a prosthetic valve, definite diagnosis of IE according to the modified Duke criteria and the 2015 ESC guidelines, *S. aureus* etiology vs. other etiologies, IE of the tricuspid valve and multiple valves vs. the aortic valve and a vegetation detected on echocardiography. In the multivariable analysis, *S. aureus* etiology vs. viridans group streptococci and enterococci, IE of the multiple valves vs. the aortic valve and a detected vegetation were associated with all embolic events.

**Table 4.** Factors associated with all embolic events during the admission in cases of infective endocarditis (*n* = 320) in Turku University Hospital in 2004-2017.

		Embolic event			
		Univariable model		Multivariable model^f^	
	Cases with embolic event (all = 125)	OR (95% CI)	P	OR (95% CI)	P
Sex^a^					
Female (*n* = 82) (%)	31 (37.8)	Reference	0.772	Reference	0.520
Male (*n* = 238) (%)	94 (39.5)	1.08 (0.64-1.84)		1.24 (0.64-2.42)	
Age group^a^			<0.001		0.549
18-39 years (*n* = 75) (%)	48 (64.0)	Reference		Reference	
40-59 years (*n* = 67) (%)	27 (40.3)	0.37 (0.18-0.73)	0.005	0.62 (0.21-1.80)	0.380
60-79 years (*n* = 134) (%)	41 (30.6)	0.24 (0.13-0.45)	<0.001	0.65 (0.23-1.82)	0.408
≥80 years (*n* = 44) (%)	9 (20.5)	0.14 (0.06-0.34)	<0.001	0.41 (0.12-1.40)	0.155
IVDU^a^					
No (*n* = 259) (%)	83 (32.0)	Reference	<0.001	Reference	0.343
Yes (*n* = 61) (%)	42 (68.9)	4.96 (2.64-9.34)		1.71 (0.57-5.15)	
Previous anticoagulation therapy^a^					
No (*n* = 236) (%)	106 (44.9)	Reference	<0.001	Reference	0.065
Yes (*n* = 84) (%)	19 (22.6)	0.36 (0.20-0.64)		0.47 (0.21-1.05)	
Coronary artery disease^a^					
No (*n* = 266) (%)	113 (42.5)	Reference	0.007	Reference	0.409
Yes (*n* = 54) (%)	12 (22.2)	0.39 (0.20-0.78)		0.71 (0.31-1.61)	
Diabetes^a^					
No (*n* = 255) (%)	106 (41.6)	Reference	0.074	Reference	0.366
Yes (*n* = 65) (%)	19 (29.2)	0.58 (0.32-1.05)		0.70 (0.32-1.53)	
Prosthetic valve^a^					
No (*n* = 260) (%)	110 (42.3)	Reference	0.019	Reference	0.239
Yes (*n* = 60) (%)	15 (25.0)	0.46 (0.24-0.88)		1.77 (0.68-4.57)	
Possible diagnosis of IE according to modified Duke criteria and ESC guidelines 2015^a^					
No (*n* = 211) (%)	100 (47.4)	Reference	<0.001	Reference	0.518
Yes (*n* = 109) (%)	25 (22.9)	0.33 (0.20-0.56)		0.79 (0.38-1.62)	
Etiology^b^			<0.001		0.026
*Staphylococcus aureus* (*n* = 109) (%)	64 (58.7)	Reference		Reference	
Viridans group streptococci (*n* = 56) (%)	14 (25.0)	0.23 (0.12-0.47)	<0.001	0.34 (0.15-0.74)	0.007
Enterococci (*n* = 27) (%)	5 (18.5)	0.16 (0.06-0.46)	<0.001	0.20 (0.06-0.63)	0.006
Coagulase-negative staphylococci (*n* = 23) (%)	5 (21.7)	0.20 (0.07-0.56)	0.003	0.41 (0.12-1.42)	0.160
Other^c^ (*n* = 63) (%)	24 (38.1)	0.43 (0.23-0.80)	0.008	0.56 (0.28-1.16)	0.117
Unknown (*n* = 39) (%)	12 (30.8)	0.31 (0.15-0.68)	0.003	0.54 (0.21-1.41)	0.206
Affected valve^a^			<0.001		0.161
Aortic only (*n* = 139) (%)	37 (26.6)	Reference		Reference	
Mitral only (*n* = 93) (%)	35 (37.6)	1.67 (0.95-2.93)	0.072	1.70 (0.91-3.21)	0.099
Tricuspid only (*n* = 45) (%)	30 (66.7)	5.50 (2.68-11.28)	<0.001	1.24 (0.46-3.34)	0.678
Multiple valves (*n* = 43) (%)	23 (53.5)	3.17 (1.57-6.38)	0.001	2.30 (1.03-5.14)	0.043
Vegetation on echocardiography^d^					
No (*n* = 78) (%)	12 (15.4)	Reference	<0.001	Reference	0.004
Yes (*n* = 240) (%)	113 (47.1)	4.88 (2.51-9.48)		3.31 (1.46-7.52)	
Heart failure during admission^e^					
No (*n* = 152) (%)	61 (40.1)	Reference	0.542		–
Yes (*n* = 161) (%)	59 (36.6)	0.87 (0.55-1.38)		–	

CI: Confidence interval; ESC: European Society of Cardiology; IE: Infective endocarditis; IVDU: Intravenous drug use; OR: Odds ratio.

^a^Cases with missing information for the analysis: 22.

^b^Cases with missing information for the analysis: 25.

^c^Other microbe than *Staphylococcus aureus*, viridans group streptococcus, enterococcus, coagulase-negative staphylococcus; or two or more microbes from the different groups mentioned.

^d^Cases with missing information for the analysis: 24.

^e^Cases with missing information for the analysis: 29.

^f^Cases with missing information for the analysis: 27.

### Cerebral embolism

Cerebral embolism was diagnosed in 51 (16.1%) patients during the IE admission (Table 5). An infected aortic valve vs. tricuspid valve was associated with cerebral embolisms in both the univariable and multivariable analysis and a detected vegetation in the multivariable analysis.

**Table 5.** Factors associated with cerebral embolisms during the admission in cases of infective endocarditis (*n* = 317) in Turku University Hospital in 2004-2017.

		Cerebral embolism			
		Univariable model		Multivariable model^d^	
	Cases with cerebral embolism (all = 51)	OR (95% CI)	P	OR (95% CI)	P
Sex^a^					
Female (*n* = 81) (%)	11 (13.6)	Reference	0.497	Reference	0.837
Male (*n* = 236) (%)	40 (16.9)	1.28 (0.62-2.64)		1.09 (0.50-2.38)	
Age group^a^			0.937		0.380
18-39 years (*n* = 73) (%)	11 (15.1)	Reference		Reference	
40-59 years (*n* = 66) (%)	11 (16.7)	1.15 (0.46-2.90)	0.769	0.47 (0.17-1.31)	0.148
60-79 years (*n* = 134) (%)	23 (17.2)	1.20 (0.54-2.69)	0.656	0.50 (0.20-1.23)	0.131
≥80 years (*n* = 44) (%)	6 (13.6)	0.92 (0.31-2.73)	0.874	0.40 (0.12-1.32)	0.132
IVDU^a^					
No (*n* = 257) (%)	41 (16.0)	Reference	0.934		–
Yes (*n* = 60) (%)	10 (16.7)	1.03 (0.47-2.28)		–	
Previous anticoagulation therapy^a^					
No (*n* = 233) (%)	40 (17.2)	Reference	0.388		–
Yes (*n* = 84) (%)	11 (13.1)	0.73 (0.36-1.49)		–	
Coronary artery disease^a^					
No (*n* = 263) (%)	46 (17.5)	Reference	0.147		–
Yes (*n* = 54) (%)	5 (9.3)	0.49 (0.18-1.29)		–	
Diabetes^a^					
No (*n* = 252) (%)	43 (17.1)	Reference	0.376		–
Yes (*n* = 65) (%)	8 (12.3)	0.69 (0.31-1.56)		–	
Prosthetic valve^a^					
No (*n* = 257) (%)	42 (16.3)	Reference	0.813		–
Yes (*n* = 60) (%)	9 (15.0)	0.91 (0.42-1.99)		–	
Possible diagnosis of IE according to modified Duke criteria and ESC guidelines 2015^a^					
No (*n* = 208) (%)	38 (18.3)	Reference	0.155		–
Yes (*n* = 109) (%)	13 (11.9)	0.61 (0.31-1.21)		–	
Etiology^b^			0.439		–
*Staphylococcus aureus* (*n* = 107) (%)	18 (16.8)	Reference			
Viridans group streptococci (*n* = 56) (%)	8 (14.3)	0.79 (0.33-1.93)	0.609	–	–
Enterococci (*n* = 27) (%)	2 (7.4)	0.36 (0.07-1.91)	0.229	–	–
Coagulase-negative staphylococci (*n* = 23) (%)	3 (13.0)	0.69 (0.18-2.73)	0.599	–	–
Other^c^ (*n* = 62) (%)	9 (14.5)	0.84 (0.37-1.92)	0.685	–	–
Unknown (*n* = 39) (%)	10 (25.6)	1.70 (0.71-4.07)	0.230	–	–
Affected valve^a^			0.139		0.046
Aortic only (*n* = 139) (%)	24 (17.3)	Reference		Reference	
Mitral only (*n* = 93) (%)	19 (20.4)	1.25 (0.64-2.44)	0.515	1.17 (0.59-2.34)	0.649
Tricuspid only (*n* = 45) (%)	1 (2.2)	0.11 (0.02-0.84)	0.033	0.05 (0.01-0.44)	0.007
Multiple valves (*n* = 40) (%)	7 (17.5)	1.01 (0.40-2.53)	0.991	0.87 (0.35-2.20)	0.773
Vegetation on echocardiography^d^					
No (*n* = 78) (%)	7 (9.0)	Reference	0.051	Reference	0.019
Yes (*n* = 237) (%)	44 (18.6)	2.34 (1.0-5.47)		2.73 (1.18-6.34)	
Heart failure during admission^e^					
No (*n* = 151) (%)	20 (13.2)	Reference	0.297		–
Yes (*n* = 160) (%)	28 (17.5)	1.39 (0.75-2.59)		–	

CI: Confidence interval; ESC: European Society of Cardiology; IE: Infective endocarditis; IVDU: Intravenous drug use; OR: Odds ratio.

^a^Cases with missing information for the analysis: 25.

^b^Cases with missing information for the analysis: 28.

^c^Other microbe than *Staphylococcus aureus*, viridans group streptococcus, enterococcus, coagulase-negative staphylococcus; or two or more microbes from the different groups mentioned.

^d^Cases with missing information for the analysis: 27.

^e^Cases with missing information for the analysis: 31.

### Pulmonary embolism

Pulmonary embolism was observed in 39 (12.4%) patients during the IE admission (cases with missing information: 27). Of the patients with pulmonary embolism, 74.4% (*n* = 29) were IVDUs. Infective endocarditis was located in the tricuspid valve in 74.4% (*n* = 29) and caused by *S. aureus* in 71.8% (*n* = 28) (cases with missing information: 30) of the patients with pulmonary embolism.

## Discussion

We described the factors associated with the different etiologies and embolic events in IE patients. We found *S. aureus* to be the most common etiology of IE (33.9% of all cases), which is in line with previous studies in different countries [6,9–12], also in Finland [8,45]. Regarding the alarming global antibiotic resistance problem, interestingly we had only one case of methicillin-resistant *S. aureus* (MRSA) (0.3% of IE cases). In another Finnish study by Halavaara et al. that analyzed IE patients from Southern Finland in 2013-2017, the proportion of MRSA was 2.2% [8]. Previous studies in Europe and Australia found the proportion of MRSA to be 4-7.5% [5,7,10,12,33]. A multicenter study involving 41 hospitals in 13 countries examining IE patients in 2015-2018 found the prevalence of MRSA to be 8.4% [11]. The finding of our study might reflect a fairly good resistance situation in our area. Furthermore, the proportion of MRSA of all *S. aureus* bacteremias in Turku University Hospital between 2005-2017 was only 1.2% (Turku University Hospital SAI-register (Sairaalan Antibiootti- ja Infektioseurantajärjestelmä, Hospital Antibiotic and Infection Monitoring System, Neotide Oy), searched April 7, 2022), which is similar to the proportion of MRSA of all *S. aureus* bacteremias found in the IE patients in this study (0.8%). Moreover, a previous population-based study found the incidence of MRSA in 2007-2016 in Hospital District of Southwest Finland to be below the national average level [46].

Enterococci caused 8.8% of the IE cases in our study. In previous studies in Finland, Halavaara et al. found the proportion of enterococci to be 9.9% [8] and Heiro et al. in their study of IE patients in Turku University Hospital in 1980-2004 the proportion of *Enterococcus faecalis* to be 8.6% [45]. Recent studies in other countries have described the proportion of enterococci as slightly higher to ours, 11.5-18% [6,11,12,33]. We found the patients with enterococcal IE to more often have heart failure (74.1%) during IE admission than the patients with other etiology. However, enterococcal IE patients were also older. Comparably, previous studies have also found patients with enterococcal IE to be older than the patients with other etiologies [19,47,48]. Furthermore, similar to the present data, a recent prospective Spanish study involving 35 centers found patients with enterococcal IE to have heart failure more frequently than the patients with IE caused by other etiology [19]. Moreover, Heiro et al. found the proportion of the *E. faecalis* IE patients with heart failure to be high, 68% [41]. In our study, enterococcal IE patients more often had a prosthetic valve (43.3%) than the patients with other etiologies. Previous studies have found enterococcal IE patients to have a prosthetic valve slightly less frequently, in 29-39% of the cases [10,17,19,49,50]. One possible reason for the higher prevalence of enterococcal IE in patients with prosthetic valves and, furthermore, for the worse clinical course of disease in patients with enterococcal IE might be that enterococci have been shown to be often present within a bacterial biofilm [51].

*Staphylococcus aureus* has been reported to be the most common causative microbe in healthcare-related IE [3,4,10]. However, enterococci have also been found to be a notable microbe causing healthcare-related IE [3,5,8,18]. In our study examining specifically the characteristics of the different microbiological etiologies, a remarkable proportion, 67.9%, of the patients with enterococcal IE had a history of a previous healthcare procedure or hospital admission, and the proportion was higher than with other etiologies. Two previous studies investigating enterococcal IE found the proportion of healthcare-associated IE to be lower, 23.4% [17] and 42.4% [19].

In the current study, the proportion of IVDUs was highest in IE caused by *S. aureus* (35.7%). Previously, of *S. aureus* IE, 21-49% has been found to be in IVDUs [23,52–54]. Our finding of the patients with *S. aureus* IE to be the youngest is likely explained by the young age of IVDUs. Furthermore, we found that the tricuspid valve was most commonly affected in *S. aureus* IE (27.8%), which is likely associated with our finding of 57.1% of IVDUs’ IE affecting the tricuspid valve. Accordingly, previously it has been described that right-sided IE is often associated with IVDU [7,8,55], and valves of the right side of the heart have been found to be affected in 8-37% of *S. aureus* IE [52–54,56–58].

We found 39.1% of the IE patients to have an embolic event during the admission. This is in line with previous studies reporting the occurrence of embolic events in 34-55% of IE cases [7,10,11,20–22]. In our study, cerebral embolism was found in 16.1% of the IE cases during admission, which is in concordance with previous studies reporting cerebral embolism in 16-26% of IE patients [7,10,11,21,22]. Heiro et al. found that 31.3% of the IE patients had embolisms outside the central nervous system and 8.3% had an embolic brain infarction [45]. Thus, the overall rate of embolic events was similar to our finding, but the occurrence of embolic brain infarction was lower than our rate of cerebral embolisms. The increased volume and furthermore accuracy of cerebral imaging over the years has likely contributed to the higher rates of embolisms detected. On the other hand, the more rapidly IE is diagnosed after the onset and antimicrobial therapy initiated combined with early surgical intervention when indicated, the less embolic events are likely to occur.

We found that IE of the multiple valves vs. the aortic valve was associated with all embolic events in the multivariable analysis. Previously, contradictory results have been demonstrated on the association of the valve localization of IE with embolic events. Some studies found the mitral valve to be associated with embolic events [31,32]. However, two multicenter studies found right-sided IE to be an independent predictor of embolic events [6,21]. A recent study in Belgium found aortic valve IE to be an independent predictor of embolic events [33]. Nevertheless, several other studies did not find any association of the valve localization of IE with embolic events [20,22,25–30].

*Staphylococcus aureus* etiology has previously been reported to be associated with an increased risk of embolic events in IE [6,21–24]. Furthermore, previous studies have found *S. aureus* etiology to be related to a higher occurrence of overall cerebral complications [39,40,59] and ischemic cerebral lesions in IE patients [36,60]. Curiously, we found that *S. aureus* etiology vs. viridans group streptococci and enterococci was associated with any embolic event, but no association with the etiology was found in cerebral embolisms. Similar results were described by Heiro et al. who found peripheral emboli to be more common in patients with *S. aureus* IE than in patients with other etiologies, whereas in cerebral embolisms there was no significant difference in the overall comparison between the different etiologies [41]. However, in their study, a multivariable analysis was not performed.

In our study, pulmonary embolism was diagnosed in 12.4% of the patients. Previously, several studies have described lower rates of pulmonary embolism, 4-9% [10,11,20–22], probably explained by the lower proportion of IVDUs (3-9%). In a recent Australian study including 23% IVDUs, pulmonary embolism occurred in 19% of the IE patients [7]. Pulmonary embolisms have previously been found to be common in IVDUs’ IEs [7,8] and to be associated with the tricuspid valve IE and *S. aureus* etiology [11,21,23,61]. Correspondingly, in our study, most of the patients with pulmonary embolism (74.4%) were IVDUs and the majority had tricuspid valve IE and *S. aureus* etiology. The importance of *S. aureus* etiology in the occurrence of all embolic events found in many studies might be at least in part explained by pulmonary embolisms. Furthermore, the proportion of pulmonary embolisms might also affect the significance of the valve localization of IE in analyses of all embolic events. To take this into account, we conducted a multivariable analysis. The youngest age group vs. other age groups, IVDU and IE of the tricuspid valve vs. the aortic valve were associated with embolic events in the univariable but not in the multivariable analysis. Nevertheless, *S. aureus* etiology vs. viridans group streptococci and enterococci was related to embolic events also in the multivariable analysis.

We found that a vegetation detected on echocardiography was associated with a higher rate of all embolic events and cerebral embolisms, which is in line with previous studies [6,31,41]. Interestingly, this was the only factor associated with both all embolic events and cerebral embolisms. Besides the vegetation, the only factor associated with cerebral embolisms was IE of the aortic valve vs. the tricuspid valve. Previously, an affected mitral valve in IE patients has been reported to be associated with cerebral complications by multiple studies [34–39]. However, some contradictory findings also exist. A Spanish multicenter study investigating neurologic complications of IE found that IE of the mitral valve or mitral and aortic vs. the aortic valve was not associated with a higher risk of ischemic neurological complications [59]. Furthermore, a recent study in France described that the aortic or mitral valve location of IE was not associated with an increased risk of neurological complications [40]. Moreover, Heiro et al. stated that there was no difference in the valve involvement of IE in the occurrence of cerebral embolisms [41]. The reason for the association of IE of the multiple valves and *S. aureus* etiology with any embolic events but not cerebral embolisms in our study remains unclear.

Our study has some limitations. It was retrospective, and some patient data were missing. However, the percentage of cases with missing information within each variable was relatively small (less than 10%). Furthermore, due to the retrospective setting, the data were recorded by treating clinicians, and there might be some errors. Some information of particular interest, for example the size of a vegetation, which is known to be associated with embolic events, was inadequately available in the patient record and was not possible to utilize in the study. Furthermore, some silent embolisms without symptoms were likely not detected in this study, as imaging was not performed in all patients. For the diagnosis of IE, we followed the modified Duke criteria and the 2015 ESC guidelines for the management of IE [1,43]. However, recently the diagnostic criteria for IE have been updated [62,63]. In this study, we did not specifically investigate surgery and did not have information on how many patients fulfilled indications for surgery but did not undergo surgery, nor when in the course of infection surgery was performed. These are factors that might have an influence on the occurrence of embolisms. Moreover, Turku University Hospital is a tertiary referral hospital and the IE patients with the most severe course of disease, and the need for surgery are transferred to this hospital from the surrounding area. Therefore, the referral bias is possible. However, in the area of the Hospital District of Southwest Finland (population approximately 480 000), practically all IE patients are treated in Turku University Hospital, and the number of patients in this study actually transferred to this hospital from outside of the Hospital District of Southwest Finland was rather small. The strength of our study is that it provides novel information on the characteristics of the different etiologies of IE and the factors, especially regarding the etiology and valve involvement, associated with all embolic events and cerebral embolisms.

In conclusion, our study suggests that enterococcal IE is often associated with a previous healthcare procedure or hospital admission and heart failure. *Staphylococcus aureus* etiology and IE of the multiple valves are associated with a higher rate of all embolic events but not cerebral embolisms. Detected vegetation is associated with a higher occurrence of embolisms.

## Data Availability

Legal restrictions apply for availability of the study data. Data are available from the authors with the permission of the Wellbeing Services Country of Southwest Finland (www.varha.fi).

## Abbreviations

CI: Confidence interval
CIED: Cardiac implantable electronic device
CT: Computed tomography
ESC: European Society of Cardiology
FAA: Fastidious anaerobe agar
ICD-10: International Classification of Diseases, Tenth Revision
IE: Infective endocarditis
IQR: Interquartile range
IVDU: Intravenous drug user or intravenous drug use
Maldi-TOF MS: Matrix-assisted laser-desorption ionization time-of-flight mass spectrometry
MRI: Magnetic resonance imaging
MRSA: Methicillin-resistant *Staphylococcus aureus*
OR: Odds ratio
PCR: Polymerase chain reaction
PET/CT: Positron emission tomography integrated with computed tomography
rRNA: Ribosomal ribonucleic acid
SD: Standard deviation
TEE: Transesophageal echocardiography
TTE: Transthoracic echocardiography
